# Protective effect of *Saussurea involucrata* polysaccharide against skin dryness induced by ultraviolet radiation

**DOI:** 10.3389/fphar.2023.1089537

**Published:** 2023-01-17

**Authors:** Lusheng Wang, Kaiye Yang, Rongrong Jing, Wengang Zhao, Keke Guo, Zhenlin Hu, Guangrong Liu, Nuo Xu, Jungang Zhao, Li Lin, Shuang Gao

**Affiliations:** ^1^ School of Pharmaceutical Sciences, Wenzhou Medical University, Wenzhou, China; ^2^ Infinitus (China) Company Ltd., Guangzhou, China; ^3^ College of Life and Environmental Sciences, Wenzhou University, Wenzhou, China; ^4^ Chevidence Lab of Child and Adolescent Health, Children’s Hospital of Chongqing Medical University, Chongqing, China

**Keywords:** *Saussurea involucrata* polysaccharide, utraviolet radiation, skin dryness, keratinocytes, PPAR-α

## Abstract

**Background:** Exposure to ultraviolet B (UVB) radiation can damage the epidermis barrier function and eventually result in skin dryness. At present, little work is being devoted to skin dryness. Searching for active ingredients that can protect the skin against UVB-induced dryness will have scientific significance.

**Methods:** *Saussurea involucrata* polysaccharide (SIP) has been shown to have significant antioxidant and anti-photodamage effects on the skin following UVB irradiation. To evaluate the effect of SIP on UVB-induced skin dryness ex vivo, SIP-containing hydrogel was applied in a mouse model following exposure to UVB and the levels of histopathological changes, DNA damage, inflammation, keratinocyte differentiation, lipid content were then evaluated. The underlying mechanisms of SIP to protect the cells against UVB induced-dryness were determined in HaCaT cells.

**Results:** SIP was found to lower UVB-induced oxidative stress and DNA damage while increasing keratinocyte differentiation and lipid production. Western blot analysis of UVB-irradiated skin tissue revealed a significant increase in peroxisome proliferator-activated receptor-α (PPAR-α) levels, indicating that the underlying mechanism may be related to PPAR-α signaling pathway activation.

**Conclusions:** By activating the PPAR-α pathway, SIP could alleviate UVB-induced oxidative stress and inhibit the inflammatory response, regulate proliferation and differentiation of keratinocytes, and mitigate lipid synthesis disorder. These findings could provide candidate active ingredients with relatively clear mechanistic actions for the development of skin sunscreen moisturizers.

## 1 Introduction

The skin is the largest organ of the human body and its main function is to protect the body from all types of external injuries. The epidermis, which is located in the skin’s outermost layer, primarily serves as the physical barrier of the skin by protecting against the admission of foreign substances and the excessive loss of endogenous materials, particularly water. Ultraviolet radiation is a critical external element that can cause excessive water loss from the skin, leading to skin dryness. Compared with UVA, UVB targets epidermal cells and causes more serious skin damage than that caused by UVA (Wang et al., 2019), with the damage extending to severe oxidative stress, DNA damage, and epidermal barrier dysfunction.

The epidermis is a stratified epithelial tissue made up of four layers, and these are the basal layer, spinous layer, granular layer, and stratum corneum. The epidermis maintains the necessary water content in the skin under physiological conditions (Wikramanayake et al., 2014). The primary constituent cells of the epidermis are the keratinocytes. These cells migrate outward from the basal layer and gradually differentiate into spinous and granular cells, which are involved in the epidermal barrier’s formation (Fuchs and Raghavan, 2002). Differentiation is associated with the regular expression of particular proteins. Involucrin and other early differentiation markers are predominantly expressed in the spinous layer, whereas loricrin and filaggrin are predominantly expressed in the granular layer (Furue, 2020). In the terminal differentiation stage, these differentiation-related proteins are highly cross-linked to form a cornified envelope (CE) under the catalysis of transglutaminase (TG), which strengthens the flexibility and mechanical elasticity of the epidermal cells, providing the correct binding sites for intercellular lipids (Candi et al., 2005). UVB impairs the function of the epidermal barrier by suppressing the expression of differentiation markers such as involucrin and filaggrin (Tu et al., 2020). As structural lipids, intercellular lipids can collaborate with the differentiated keratinocytes to maintain the epidermal barrier. Lamellar bodies are organelles found in the granular layer of keratinocytes that store and transport lipids. When lipids are synthesized, lamellar bodies transport them from the granular layer cells to the extracellular space of the stratum corneum to constitute intercellular lipids (Feingold and Elias, 2014). Intercellular lipids are composed of 50% ceramides, 25% cholesterol, 15% free fatty acids, and a trace amount of other lipids (Feingold, 2007; Olivier et al., 2017). Cholesterol has been proven to regulate keratinocyte differentiation (Vietri Rudan and Watt, 2021). Cholesterol synthesis increases with keratinocyte differentiation and plays an important role in the formation of lamellar bodies. Long-chain fatty acids can be synthesized by the extra-long-chain fatty acid elongases (ELOVLs) (Ehehalt et al., 2006). Furthermore, UVB-induced aberrant lipid metabolism can also exacerbate the failure of the epidermal barrier, all of which can eventually contribute to skin dryness (Takagi et al., 2004; Bak et al., 2011).

Skin dryness can be prevented by various products, some of which are derived from natural products, such as plant polysaccharides. *Saussurea involucrata* is a perennial herb related to compositae and chamomile, and *saussurea involucrata* polysaccharide (SIP) has recently been found to be capable of scavenging a wide variety of reactive oxygen free radicals (Yao et al., 2012; Chen et al., 2018). These data suggest that SIP may have a role in dermatology, particularly in photoprotection. The objective of this study was to evaluate whether SIP has a protective effect on skin dryness induced by UVB and to explore the underlying mechanism associated with such effect *via* the perspectives of DNA damage and oxidative stress, abnormal proliferation and differentiation of keratinocytes, and reduction of epidermal intercellular lipid production.

## 2 Materials and methods

### 2.1 Materials

*Saussurea involucrata* polysaccharide was provided by Infinitus Co., Ltd. (Guangzhou, China). PPAR-α inhibitor (GW6471) was purchased from Selleck (Shanghai, China).

### 2.2 Determination of molecular weight

The homogeneity and molecular weight of SIP were measured using a gel permeation chromatography-eighteen angles laser light scattering instrument (GPC-MALLS) (Wyatt Technology Corporation, Santa Barbara, CA, United States). In brief, 10 mg of SIP was placed in 1 mL of .1 M NaNO_3_ solution in a small beaker, which was then placed on a magnetic stirrer (Crystal Technology & Industries, Dallas, TX, United States) and stirred overnight to dissolve the SIP. The SIP sample was diluted fourfold with .1 M NaNO_3_ solution and then filtered through a .22-μm nylon filter (Millipore Corp., Billerica, MA, United States). The filtered sample was then subjected to gel permeation chromatography using a GPC-MALLS system. The samples were resolved by three tandem columns (300 × 8 mm, Shodex OH-pak SB-805, 804 and 803; Showa Denko K.K., Tokyo, Japan) with .1 M NaNO_3_ solution used as a mobile phase under a flow rate of .4 mL/min. The data were collected and analyzed with the Astra 6.1 software (Wyatt Technology Corporation, Santa Barbara, CA, United States).

### 2.3 Analysis of monosaccharides

The monosaccharide composition of SIP was analyzed using high-performance anion-exchange chromatography (HPAEC). In brief, 1 mL of 2 M TFA was added to 5 mg of polysaccharide sample, and the mixture was incubated in a 121°C oil bath for 2 h. The sample was soaked in methanol and blown dry with nitrogen to remove residual TFA, and then freeze-dried. The residue was re-dissolved in deionized water and filtered through a .22-μm microporous membrane filter for HPAEC analysis. The sample extracts were analyzed by high-performance anion-exchange chromatography (HPAEC) using a CarboPac PA-20 anion-exchange column (3 by 150 mm; Dionex) connected to a pulsed amperometric detector (PAD; Dionex ICS 5000 system). The column was run at a flow rate of .5 mL/min. Data were acquired by an ion chromatography system (ICS5000, Thermo Scientific) and processed using chromeleon 7.2 CDS (Thermo Scientific).

### 2.4 Hydrogel preparation

To make the hydrogel for topical application, .6 g carbopol, 5 g glycerinum, and 3 g propylene glycol were mixed in 20 g of water and slowly stirred until to obtain a uniform suspension. Next, .6 g of trietha-nolamine (TAE) was added to the suspension and with slow stirring to produce a hydrogel matrix. To make the SIP hydrogel, .1 or .5 g of SIP was dissolved in 80 g of water, and the SIP solution was slowly added to the hydrogel matrix to generate a homogenous mixture containing either .1% or .5% SIP. These SIP-containing hydrogel preparations will henceforth be referred to as .1% SIP hydrogel and .5% SIP hydrogel.

### 2.5 UVB-induced skin dryness model and treatments

Healthy male C57BL/6 mice (seven-week-old, 18–22 g body weight) were purchased from Gempharmatech Co., Ltd. (Jiangsu, China). The mice were kept at 22°C ± 1°C with 50%–55% relative humidity and a 12-h light/dark cycle. After 1 week of acclimatization, the hairs on the dorsal skin were shaved and the mice were then randomly divided into four groups. One group was designated as the healthy control group, and it received no treatment. The other three groups were treated with UVB, and one of these groups was treated with hydrogel only, whereas the other two groups were each treated with either the .1% or .5% SIP hydrogel. Hydrogel was completely wiped from the dorsal skin of mice prior to UVB irradiation, and then the animals were exposed to UVB irradiation at a dose of 500 mJ/cm^2^ for five consecutive days and the daily transdermal water loss (TEWL) was determined using a VapoMeter (Delfin Technologies, Finland). Besides, a cuticle hydration meter (MoistureMeter Epid, Delfin Technologies, Finland) was used to determine epidermal water content. During these 5 days of exposure to UVB, hydrogel or SIP hydrogel was applied to the dorsal skin twice daily, and the skin was photographed with a camera prior to the daily UVB exposure.

All animal experiments were conducted according to international ethical guidelines and the National Institutes of Health Guide for the Care and Use of Laboratory Animals, and all protocols were approved by the Animal Care and Use Committee of Wenzhou Medical University.

### 2.6 Histological examination

The fixed slices were treated with 3% H_2_O_2_ and then blocked with 5% BSA. The specimens were finally incubated with anti-Filaggrin (Santa Cruz, California, United States) or anti-Involucrin (1: 500; Absin, Shanghai, China) antibody overnight at 4°C, and then incubated with horseradish peroxidase-conjugated secondary antibody at 37°C for 4 h followed by reaction with 3,3-diaminobenzidine (DAB).

### 2.7 Immunofluorescence staining

The slices were first treated with 3% H_2_O_2_ and then blocked with 5% BSA*.* Next, the slices were treated with the proliferation marker Ki67 (Cell Signaling Technology, Beverly, MA, United States) overnight at 4°C. After that, the slices incubated with Alexa Flour 488 anti-rabbit secondary antibody at 37°C for 1 h. The immunofluorescence assays for *γ*-H2AX and CPD used *γ*-H2AX (1:1,000) and anti-CPD (1:200) (Cell Signaling Technology, Beverly, MA, United States) as primary antibodies. The immunofluorescence assays for K16 used Keratin 16 (1:500) (Proteintech, Chicago, IL, United States) as primary antibody. Finally, samples were stained with antifade reagent containing DAPI (Invitrogen, Life Technologies, California, United States) and examined with confocal laser scanning microscope (Olympus, Tokyo, Japan).

### 2.8 Nile red fluorescence staining

The skin samples were embedded in Tissue Tek OCT medium (Sakura, Tokyo, Japan) and stored at −80°C until completely solidified, after which the samples were cut into 10-μm slices. The slices were incubated with Hoechst solution (Dojindo, Tokyo, Japan) and then in Nile Red Fluorescent Dye Working Solution (Glpbio, Montclair, CA, United States). Finally, the samples were placed in glycerin gelatin and examined under confocal laser scanning microscope.

### 2.9 UVB irradiation

HaCaT cells were treated without or with SIP (5, 10, 20, and 50 μg/mL) for 24 h, and then washed with PBS. The cells were then exposed to UVB (200 mJ/cm^2^) in PBS using a VL6-M Biotronic device (Vilber Lourmat, Marne La Vallee, France). After UVB exposure, the cells were incubated with fresh DMEM at 37°C in a 5% CO_2_ incubator for differenttimes depending on the experiment.

### 2.10 Intracellular reactive oxygen species measurement

HaCaT cells were treated without or with SIP (5, 10, 20, and 50 μg/mL) for 24 h followed by exposure to UVB (200 mJ/cm^2^) in PBS. After that, the cells were stained with annexin V & FITC apoptosis detection kit (Dojindo, Tokyo, Japan) and then quantified with a flow cytometer equipped with an ACEA NovoCyte (Agilent, Santa Clara, CA, United States).

### 2.11 Immunofluorescence staining for HaCaT cells

HaCaT cells were pretreated without or with SIP (5, 10, 20, 50 μg/mL) for 24 h, followed by exposure to UVB (200 mJ/cm^2^) in PBS. After washing with PBS, the cells were incubated for 6 h in DMEM, fixed in 4% paraformaldehyde and permeabilized in .3% Triton X-100. This was followed by blocking with 5% BSA-and overnight incubation with *γ*-H2AX and anti-CPD or Ki67 at 4°C. After that, the cells were finally treated with an appropriate secondary antibody and mounted with an antifade reagent.

### 2.12 Calcein-AM/PI double staining

HaCaT cells were pretreated without or with SIP (5, 10, 20, 50 μg/mL) for 24 h, followed by exposure to UVB (200 mJ/cm^2^) in PBS. The cells were then washed with PBS and incubated for 6 h in DMEM. After that, the cells were stained with a Calcein-AM/PI double staining kit (Dojindo, Tokyo, Japan) and then examined under a fluorescence microscope.

### 2.13 Oil-red O staining quantitative assay

HaCaT cells were cultured in a 6-well plate in the presence of 10 mM Ca^2+^ for 24 h and then treated without or with SIP (5, 10, 20, 50 μg/mL) for 24 h followed by exposure to UVB (200 mJ/cm^2^). The cells were then washed with PBS and incubated for 6 h in DMEM followed by oil-red O staining using a commercial staining kit (Solarbio, Beijing, China).

### 2.14 Quantitative PCR

Total RNA from a skin sample was extracted with TRIzol^®^ Reagent (Thermo Fisher Scientific, Waltham, MA, United States) whereas total RNA form HaCaT cells was extracted with an RNA isolation kit (Biomiga, San Diego, CA, United States) according to the manufacturer’s protocols using. For each sample, 1 μg of total RNA was reverse transcribed using Prime Script RT reagent Kit (Takara, Dalian, China). Quantitative real-time PCR (qPCR) was performed on an LC96 system (Roche, Basel, Switzerland) using the SYBR Green Master Mix (Applied Biosystems, Foster City, CA). The comparative Ct approach was utilized to assay the mRNA relative levels while the endogenous control was GAPDH while the2^−ΔΔCt^ method was employed to quantify the relative target gene expression. The sequences of the primers used are shown in Table 1.

**TABLE 1:** The primers sequences for quantitative PCR (qPCR).

Gene	Species	Primer sequence (5′–3′)
IL-1β	Mouse	F: 5′-TGT​GTA​ATG​AAA​GAC​GGC​ACA​CC-3′
		R: 5′-GTA​TTG​CTT​GGG​ATC​CAC​ACT​CTC-3′
TNF-α	Mouse	F: 5′-CAG​GCG​GTG​CCT​ATG​TCT​CA-3′
		R: 5′-GGC​TAC​AGG​CTT​GTC​ACT​CGA​A-3′
GAPDH	Mouse	F: 5′-TTA​AGA​GGG​ATG​CTG​CCC​TTA​CCC-3′
		R: 5′- TTG​TCT​ACG​GGA​CGA​GGA​AAC​AC-3′
IL-1β	Human	F: 5′-ACG​AAT​CTC​CGA​CCA​CCA​CTA​C-3′
		R: 5′-TCC​ATG​GCC​ACA​ACA​ACT​GAC​G-3′
TNF-α	Human	F: 5′-TGA​GCA​CTG​AAA​GCA​TGA​TCC​G-3′
		R: 5′-AGA​AGA​GGC​TGA​GGA​ACA​AGC​AC-3′
ELOVL1	Human	F: 5′-TCG​CAT​CAT​GGC​TAA​TCG​GAA​GC-3′
		R: 5′-AGA​GTG​CCA​CCA​GTG​AGA​AGT​TG-3′
ELOVL4	Human	F: 5′-ATG​CAG​TCT​CCT​TGG​CCT​ACA​C-3′
		R: 5′-TTG​GAC​CCA​GCC​ACA​CAA​ACA​G-3′
ELOVL6	Human	F: 5′-AGG​CCT​GAA​GCA​GTC​AGT​TTG-3′
		R: 5′-AAG​CCC​AGA​ATT​TGC​TGA​CAG​G-3′
GAPDH	Human	F: 5′-TCC​TCT​GAC​TTC​AAC​AGC​GAC​AC-3′
		R: 5′-CAA​AGT​GGT​CGT​TGA​GGG​CAA​TG-3′

### 2.15 Western blot

Extracts of a skin sample or HaCaT cells were prepared and then centrifuged at 12,000 × *g* to precipitate the insoluble materials. The supernatant was retained and the concentration of protein in the supernatant was measured by a BCA Protein Assay Kit (Beyotime, Jiangsu, China). A sample of supernatant was resolved by SDS-PAGE using a 12% gel. The protein bands in the gel were transferred to a polyvinylidene difluoride (PVDF) membrane and blocked with 5% skim milk, followed by overnight incubation with anti-Filaggrin, anti-Involucrin, anti-PPARα, anti-PPARγ, anti-LXR (Proteintech, Chicago, IL, United States), or anti-ABCA1 (Absin, Shanghai, China). After that, the blot was washed and incubated with the appropriate secondary antibody (Cell Signaling Technology, Beverly, MA, United States). Finally, the blot was subjected to a detection assay using a chemiluminescence substrate (Pierce, Rockford, IL, United States), and images of the blot were acquired using an Amersham Imager (GE Healthcare Biosciences, Pittsburgh, PA, United States).

### 2.16 Statistical analysis

GraphPad Prism 6.0 software (GraphPad, San Diego, CA, United States) was used for all statistical analyses. The statistical differences between groups were performed using one-way analysis of variance (ANOVA), and correction for multiple comparisons was made using Dunnett’s test.

## 3 Results

### 3.1 Physicochemical properties of SIP

**FIGURE 1:**
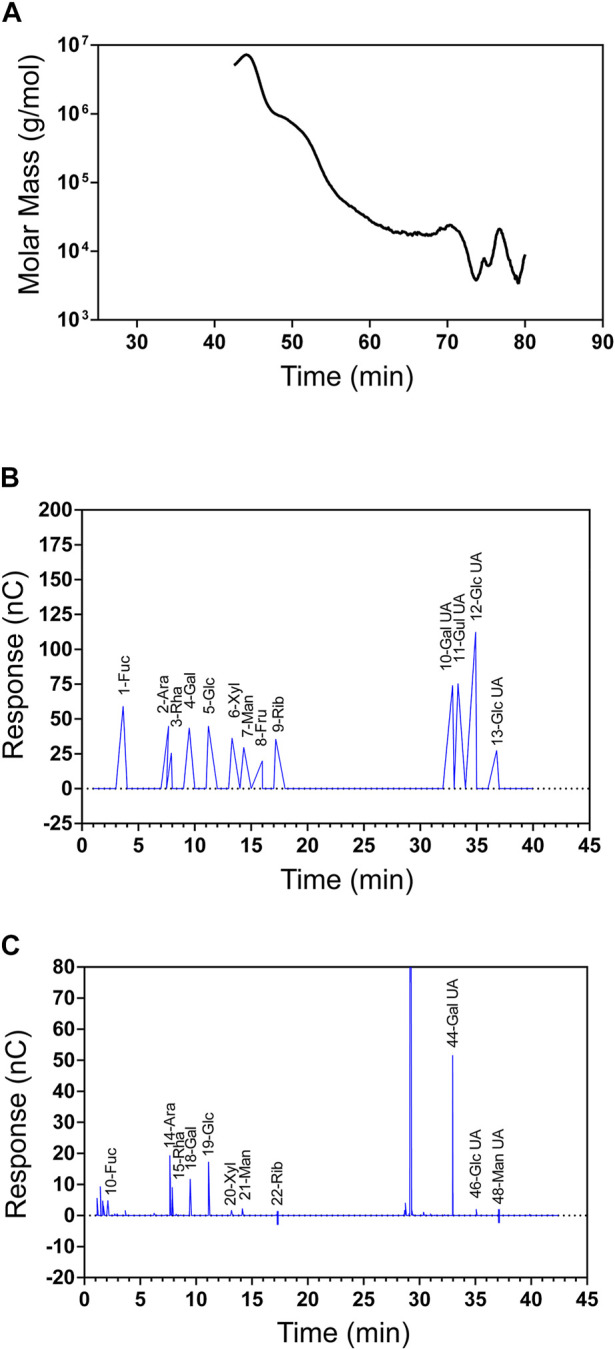
Physicochemical properties of SIP. **(A)** SIP molecular weight and homogeneity as determined by GPC-MALLS, which included a refractive index detector (Waters 2,414 (RI) and a Wyatt DAWN EOS MALLS detector. **(B)** Standard monosaccharide ion chromatogram (Fuc, Ara, Rha, Gal, Glc, Xyl, Man, Fru, Rib, Gal-UA, Gul-UA, Glc-UA, and Man-UA). **(C)** SIP ion chromatogram.

The molecular weight of SIP was determined to be 120 kDa according to GPC-MALLS analysis (Figure 1A). The HPAEC chromatogram for the mixed standard monosaccharide is shown in Figure 1B. The horizontal coordinate is the retention time (Time, min), and the vertical coordinate is the ion response value (Response, nC). According to the retention time of standard monosaccharides, SIP was found to be primarily composed of galacturonic acid, arabinose, glucose, rhamnose, and galactose (44.21: 16.37: 12.19: 10.06: 9.7). (Figure 1C).

### 3.2 SIP relieves UVB-induced epidermal injury

**FIGURE 2:**
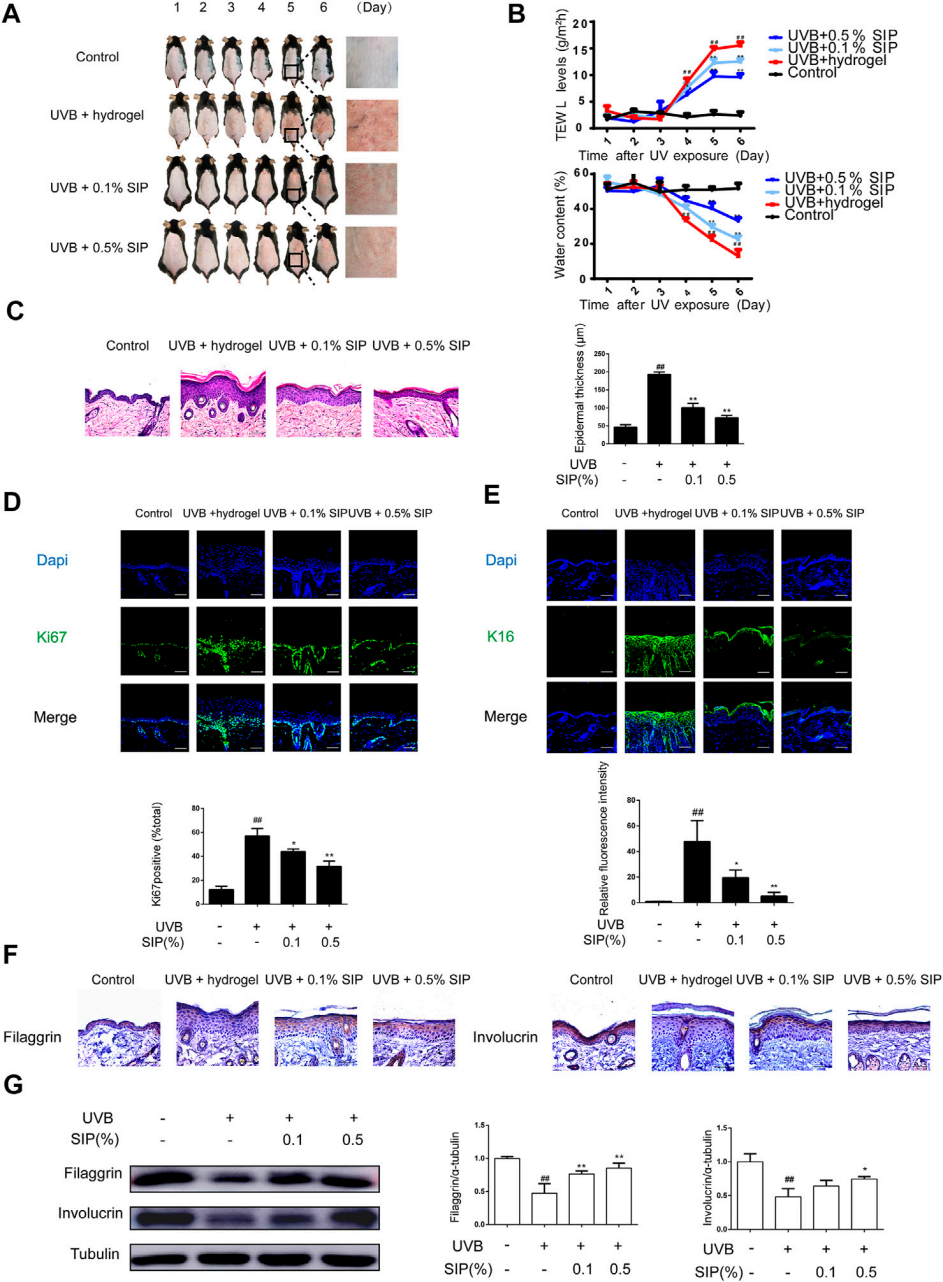
Effects of SIP hydrogel on UVB-induced epidermal dryness in mice. The first day that the shaven dorsal skin received the UVB irradiation was considered Day 1. On Day 1, hydrogel or SIP hydrogel (.1% and .5% SIP) was topically applied to the skin after UVB irradiation. The treatment given on Day 1 was repeated on the next five consecutive days (Days 2, 3, 4, and 5). Skin samples were collected from the mice on Day 6. **(A)** Morphological changes in the skin lesions were observed by photographing. **(B)** TWEL and water content values were measured at 1, 2, 3, 4, 5, and 6 days. **(C)** Histological changes in the skin lesions were observed by HE. The plots beside the images show the quantitation analysis of epidermal thickness. **(D)** Immunostaining of Ki67 protein expression in epidermal tissue. The plots under the images show the quantitation analysis of Ki67 positive cells. **(E)** Immunostaining of K16 protein expression in epidermal tissue. The plots under the images show the quantitation analysis of K16 fluorescence intensity. **(F)** Immunohistochemical analysis of the expression of filaggrin and involucrin in epidermal tissue (original magnification × 200). **(G)** Changes in the protein expression of filaggrin and involucrin as determined by western blot. The relative levels of filaggrin and involucrin were determined by densitometric analysis of the bands in the blots. Data shown in each plot are the mean ± SD of three independent experiments. “##” indicates significantly different from the control at the *p* < .01 level. “*” and “**” indicate significantly different from the UVB group at the *p* < .05 and *p* < .01 levels, respectively.

To investigate the photoprotective effect of SIP against UVB radiation and its healing effects on the recovery of the damaged epidermal barrier, the shaved dorsal skin of mice was exposed to repeated UVB radiation followed by topical application of hydrogel without or with SIP. Compared with the UVB group, SIP hydrogel application dramatically reduced dryness, peeling, scaling, and erythema caused by UVB radiation (Figure 2A). Additionally, decreased UVB-induced trans epidermal water loss was also evident as revealed by TWEL measurement (Figure 2B). After repeated UVB irradiation, HE staining of skin samples revealed abnormal epidermal thickening and the development of hyperplasia-like symptoms. The epidermis thickness was considerably lowered by increasing the dose of the SIP hydrogel applied (Figure 2C). Immunofluorescence analysis of the epidermal cells in the proliferation cycle tagged with the proliferation-related antigen Ki67 revealed a substantially higher number of Ki67-labeled cells in the UVB group compared with the healthy control group. However, for SIP hydrogel group, the number of Ki67-labeled cells was significantly reduced (Figure 2D). Abnormal proliferation of epidermal cells usually leads to epidermal hyperplasia. To further investigate the effect of SIP on epidermal hyperplasia induced by UVB, the marker of epidermal hyperplasia K16 was stained. Immunofluorescence analysis showed that compared with the UVB group, SIP hydrogel treatment significantly reduced the fluorescence intensity of k16 in epidermal, suggesting that SIP could effectively prevent UVB-induced epidermal hyperplasia (Figure 2E). The dynamic balance of keratinocyte proliferation and differentiation maintains the epidermal barrier, which is necessary for skin moisturization. To test if SIP could hasten the epidermal barrier repair process following UVB exposure, the expression levels of the keratinocyte differentiation markers, filaggrin and involucrin were measured by immunohistochemistry and western blot. The expression of these proteins in the epidermi*s* was inhibited by UVB, but the inhibition was attenuated following the application of SIP hydrogel (Figures 2F, G)*.* SIP kept the skin moist following UVB-irradiation by reducing UVB-induced dehydration and hyperplasia of the epidermis, enabling it to prevent or reduce the extent of UVB-induced disorders in epidermal proliferation and differentiation, thereby assisting with the recovery of the barrier function.

### 3.3 SIP alleviates UVB-induced epidermal DNA damage and inflammatory response

**FIGURE 3:**
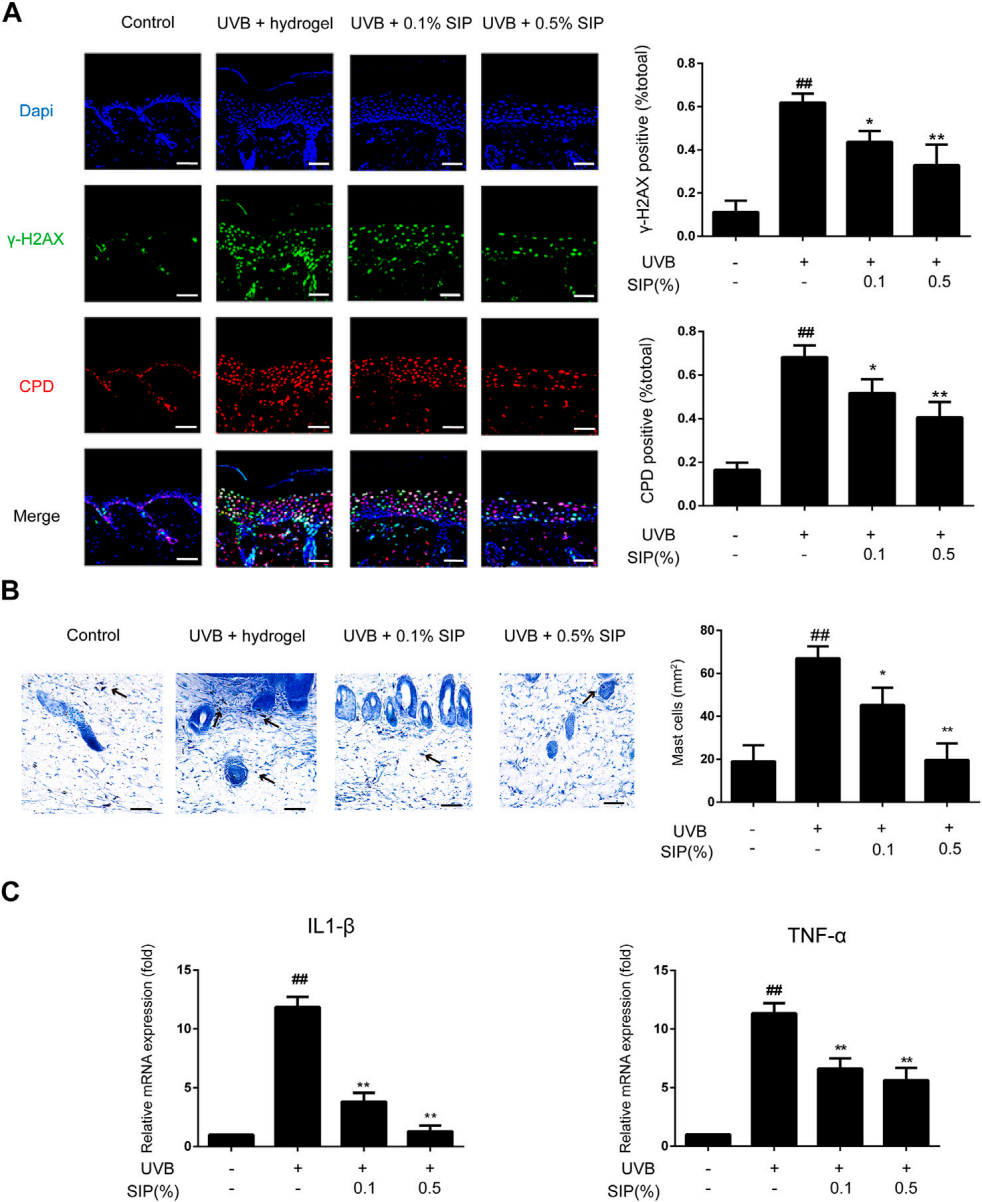
Effects of SIP hydrogel on UVB-induced epidermal DNA damage and inflammation. **(A)** Changes of *γ*-H2AX and CPD in the epidermis as examined by immunofluorescence staining. The plots beside the images show the quantitation analysis of *γ*-H2AX and CPD positive cells. **(B)** Histological changes in the skin lesions were observed by Mast Cells Staining. The plots beside the images show the quantitation analysis of mast cell numbers. **(C)** Changes in the mRNA expression of pro-inflammatory factors (IL-1β, TNF-α) on mouse skin samples by qPCR. Data shown in each plot are the mean ± SD of three independent experiments.

Another major mechanism for UVB-induced skin dryness is the induction of oxidative stress, which can damage cellular DNA and trigger severe inflammatory reactions. The degree of DNA damage can be evaluated by measuring the level of CPD, the major photoproduct of UVB-induced DNA damage, and *γ*-H2AX, a DNA damage-sensing molecule. Compared with the healthy control group, a significantly higher *γ*-H2AX and CPD content was found in the UVB group, where the two markers were present in 60% of the epidermal cells. However, both *γ*-H2AX and CPD contents were significantly reduced in the .1% or .5% SIP hydrogel group, where only 40% of the epidermal cells displayed the two markers (Figure 3A). Many inflammatory lesions are characterized by abnormal aggregation and activation of mast cells, which will release many cytokines such as IL-1 and TNF-α as a result of their own activation, leading to chronic inflammation. UVB radiation led to abnormal aggregation of mast cells in the skin, while mast cell aggregation was significantly reduced in the SIP hydrogel group (Figure 3B). Furthermore, SIP hydrogel application also lowered the mRNA levels of the pro-inflammatory factors IL-1β and TNF-α in a dose-dependent manner (Figure 3C). Taken together, the findings indicated that SIP could protect the skin against UVB-induced DNA damage while decreasing UVB-induced inflammatory responses.

### 3.4 SIP mitigates UVB-induced skin dryness by promoting epidermal lipid synthesis

**FIGURE 4:**
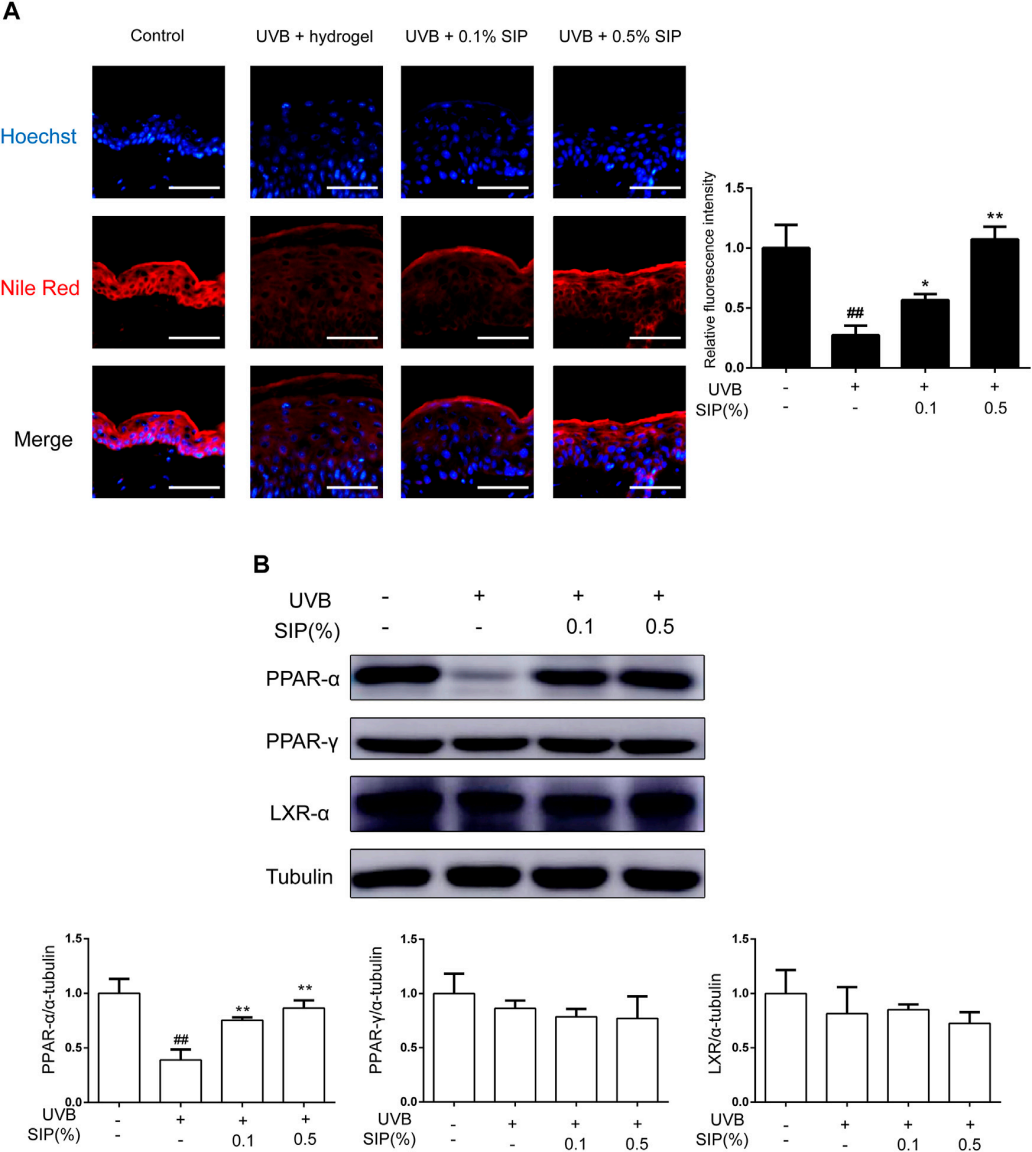
Effects of SIP hydrogel on the epidermal lipid synthesis. **(A)** Changes in neutral lipid of the epidermis as examined by Nile red fluorescence staining. The plots beside the images show the quantitation analysis of neutral lipids fluorescence intensity. **(B)** Changes in the protein expression of PPAR-α, PPAR-γ, and LXR-α as revealed by western blot. The relative levels of PPAR-α, PPAR-γ, and LXR-α were determined by densitometric analysis of the bands in the blots. Data shown in each plot are the mean ± SD of three independent experiments.

Disorder in the synthesis of epidermal lipids can lead to the loss of the epidermal permeability barrier, which will consequently interfere with the ability of the skin to maintain its moisture and lead to skin dryness. The majority of epidermal intercellular lipids are neutral lipids, and the metabolism of neutral lipids is essential to the modulation of the skin permeability barrier. Nile red is a lipophilic fluorescent dye that is typically used to identify neutral lipids. A lower content of neutral lipids was found in the epidermis of the UVB group relative to that of the healthy control group, whereas for the SIP hydrogel group, the content of neutral lipids was comparable with that of the healthy control group (Figure 4A). This suggested that SIP could prevent the loss of epidermal intercellular lipid induced by UVB, thereby, maintaining the epidermal barrier. Peroxisome proliferator-activated receptors (PPARs) and liver X receptors (LXRs) are important regulators of keratinocyte proliferation and differentiation, as well as lipid synthesis. UVB radiation inhibits the expression of PPAR-α in the epidermis, and this may be the underlying mechanism for UVB-induced skin dryness. SIP antagonized the inhibitory effect of UVB on PPAR-α protein as shown by western blot analysis (Figure 4B). These data indicated that SIP might contribute to mitigating effect on UVB-induced skin dryness through the inhibition of disorder in epidermal lipid synthesis, the regulation of epidermal proliferation and differentiation, and the regulation of lipid synthesis *via* PPAR-α.

### 3.5 SIP relieves UVB-induced keratinocytes damage

**FIGURE 5:**
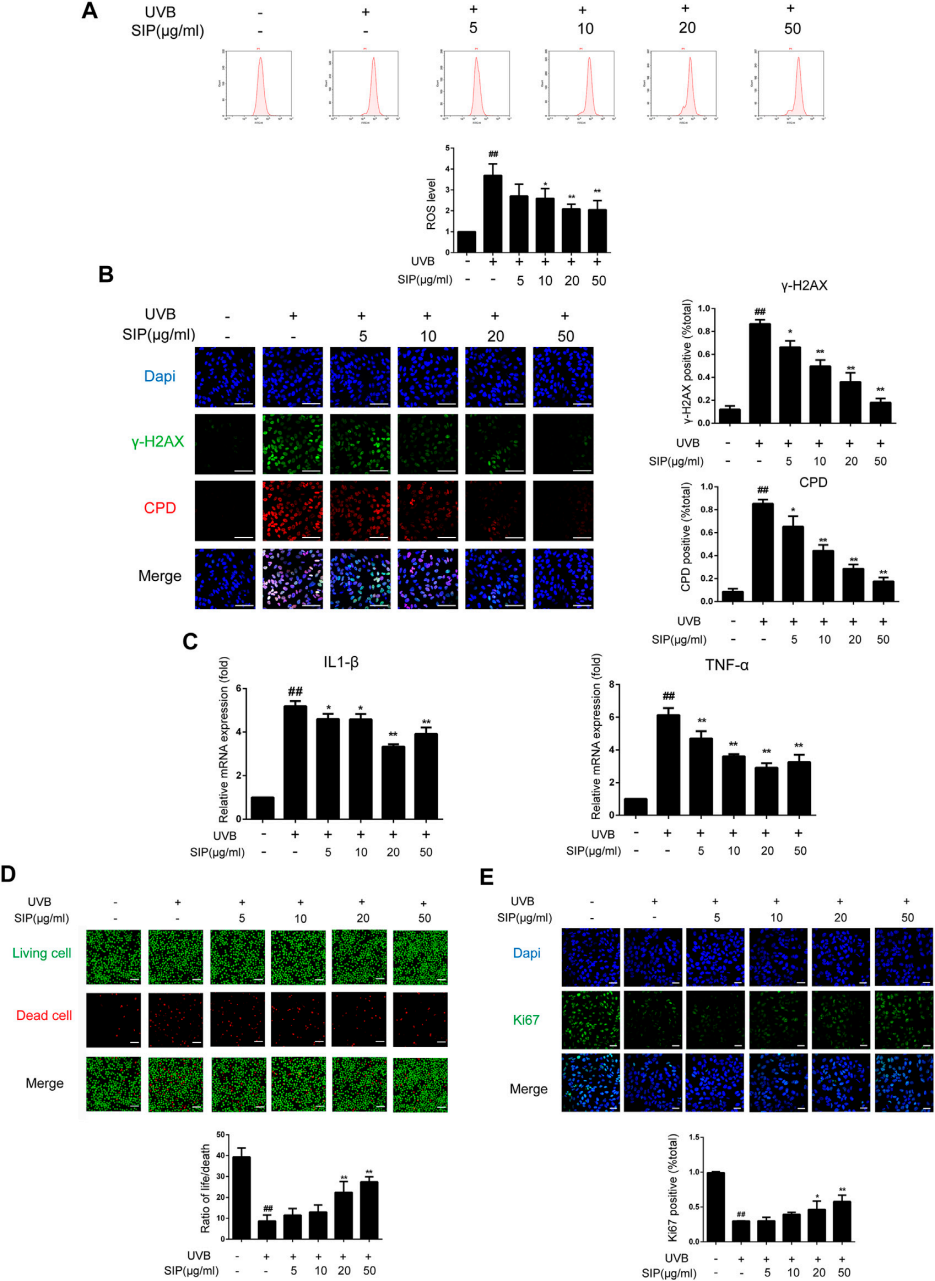
Effects of SIP on UVB-induced keratinocyte damage. HaCaT cells were treated with various concentrations (5, 10, 20, and 50 μg/mL) of SIP for 24 h, and then irradiated with UVB (200 mJ/cm^2^) and cultured for 6 h **(A)** ROS levels in HaCaT cells were detected by flow cytometry. The plots below the images show the quantitation analysis of ROS levels. **(B)** Changes in *γ*-H2AX and CPD levels in HaCaT cells as examined by immunofluorescence staining. The plots beside the images show the quantitation analysis of *γ*-H2AX and CPD positive cells. **(C)** Changes in the mRNA expression levels of pro-inflammatory factors (IL-1β, TNF-α) in HaCaT cells as measured by qPCR. **(D)** Changes in HaCaT cell death as detected by Calcein-AM/PI fluorescence double staining. The plots below the images show the ratio of life and dead cells. **(E)** Changes in the proliferation marker Ki67 in HaCaT cells as examined by immunofluorescence staining. The plots below the images show the quantitation analysis of Ki67 positive cells. Data shown in each plot are the mean ± SD of three independent experiments. “##” indicates significantly different from the control at the *p* < .01 level. “*” and “**” indicate significantly different from the UVB alone at the *p* < .05 and *p* < .01 levels, respectively.

Oxidative stress is the most direct injury sustained by keratinocytes following exposure to UVB irradiation, and ROS are byproducts of UV-absorbing cells. The production of ROS and the occurrence of oxidative stress in HaCaT cells induced by UVB irradiation were both reduced following treatment with SIP as revealed by flow cytometry (Figure 5A). In line with the ROS results, SIP treatment reduced the *γ*-H2AX and CPD content of UVB-irradiated HaCaT cells in a dose-dependent manner (Figure 5B). Furthermore, qPCR results of IL-1β and TNF-α showed that SIP reversed the UVB-induced upregulation of mRNA levels of pro-inflammatory factors, indicating that SIP could relieve the UVB-induced inflammatory response in HaCaT cells (Figure 5C). Ultraviolet radiation has a cytotoxic effect on skin cells, leading to cell death. The use of calcein-AM/PI fluorescence staining on HaCaT cells revealed more dead cells after UVB radiation and a lower proportion of life/death ratio compared with the healthy control group (Figure 5D). Treatment with a high dose of SIP (20, 50 μg/mL) was found to lower the number of dead cells while increasing the life/death ratio by more than twofold. Besides, Ki67 fluorescence staining revealed a lower number of Ki67-labeled cells for the UVB irradiated cells compared to the non-treated cells. However, upon SIP treatment, the number of Ki67-labeled cells increased for the UVB irradiated cells (Figure 5E). Overall, SIP could reduce UVB-induced keratinocyte damage, including oxidative stress and inflammation responses, thereby preventing cell death and protecting cell proliferation-associated activity.

### 3.6 SIP mitigates UVB-induced keratinocytes lipids syntheses dysfunction

**FIGURE 6:**
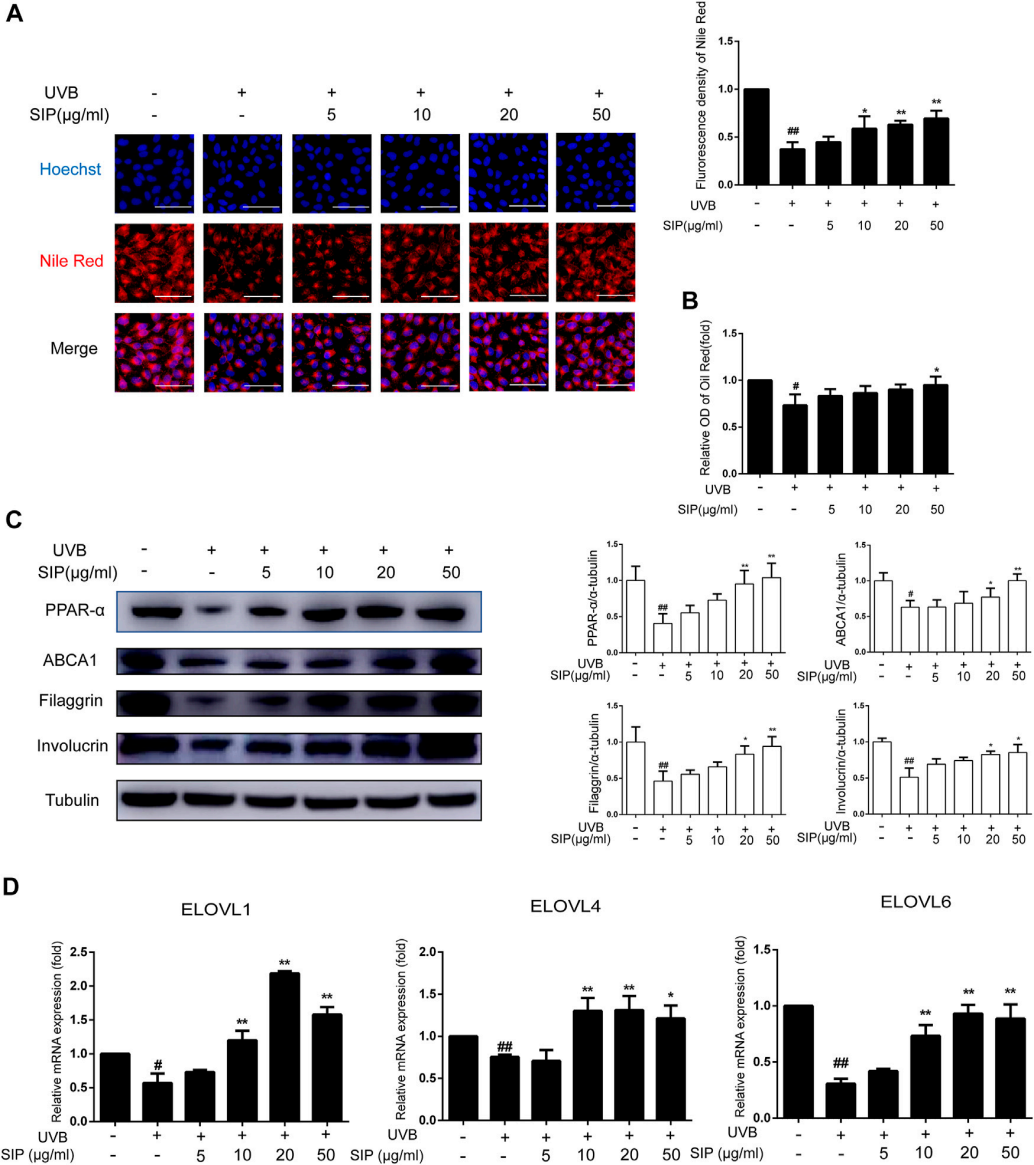
Effects of SIP on keratinocyte lipid synthesis. **(A)** Changes in neutral lipid level of HaCaT cells as examined by Nile red fluorescence staining. The plots beside the images show the quantitation analysis of neutral lipids fluorescence intensity. **(B)** Detection of lipid content in HaCaT cells by oil-red O. **(C)** Changes in the expression of PPAR-α, involucrin, filaggrin, and ABCA1 by western blot. The relative levels of PPAR-α, involucrin, filaggrin, and ABCA1 were determined by densitometric analysis of the bands in the blots. **(D)** Changes in the mRNA levels of ELOVL1, ELOVL4, and ELOVL6 in HaCaT cells as measured by qPCR. Data shown in each plot are the mean ± SD of three independent experiments.

To elucidate the mechanism by which SIP might have prevented the UVB-induced disorder in epidermal intercellular lipid synthesis, the neutral lipid content in HaCaT cells was examined by Nile red fluorescence and oil-red O staining. Both staining revealed reduced lipid content in HaCaT cells following UVB irradiation, but the irradiated cells that were also treated with SIP displayed a normal level of lipid content (Figures 6A, B). Activation of the PPAR-α pathway has previously been shown to increase keratinocyte differentiation and boost lipid production, thus increasing epidermal barrier function. Therefore, we examined the expression levels of PPAR-α and its downstream target genes filaggrin, involucrin, and ABCA1. UVB was found to inhibit the protein expression of PPAR-α and its target genes, but SIP could prevent this effect (Figure 6C). ELOVLs are key enzymes in the synthesis of long-chain fatty acids. Compared with the healthy control group, UVB treatment caused a reduction of ELOVL1, ELOVL4, and ELOVL6 mRNA levels in the cells, but UVB + SIP treatment not only prevented the loss of these mRNA levels (Figure 6D). High concentrations of SIP also increased the mRNA levels of ELOVL1 and ELOVL4 beyond the levels of the control. The result indicated that SIP could abolish the inhibitory effect of UVB on the expression of genes involved in fatty acid synthesis. SIP could prevent the occurrence of keratinocyte differentiation and lipid synthesis disorder induced by UVB, and the PPAR-α signaling pathway may be involved in the overall protective effect of SIP against UVB-induced skin dryness.

### 3.7 PPAR-α is involved in the epidermal barrier regulatory function of SIP

**FIGURE 7:**
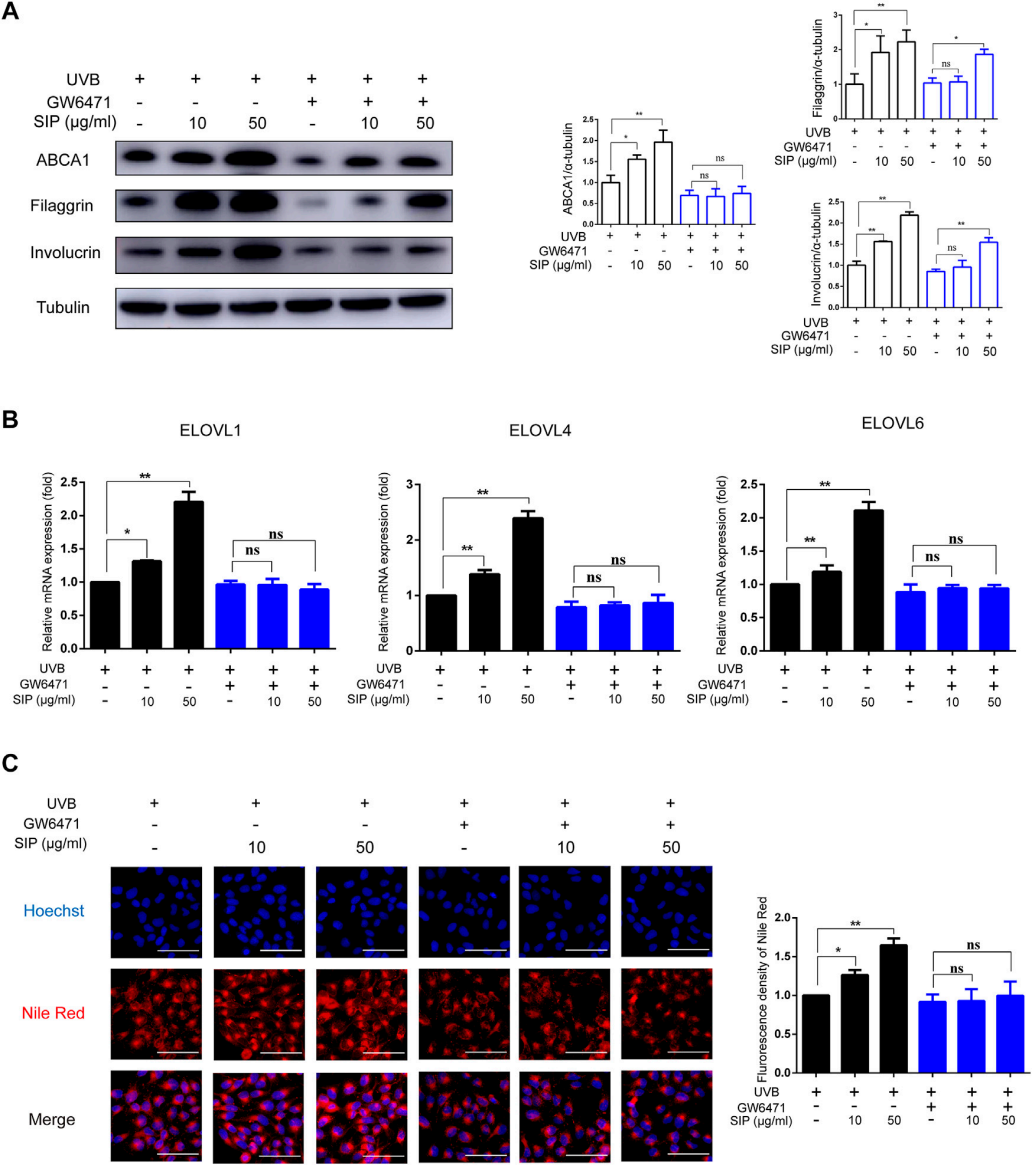
Effects of SIP on UVB-induced inhibition of keratinocyte differentiation and lipid synthesis. HaCaT cells were cultured with 10 mM Ca^2+^ for 24 h, and then treated with SIP (10, 50 μg/mL) in the presence or absence of GW6471 (.2 μM) for 24 h and irradiated with UVB (200 mJ/cm^2^). **(A)** Changes in the protein expression of ABCA1, involucrin and filaggrin as detected by western blot. The relative levels of ABCA1, involucrin and filaggrin were determined by densitometric analysis of the bands in the blots. **(B)** Changes in the mRNA expression of ELOVLs in HaCaT cells by qPCR. **(C)** The level of neutral lipid was examined by Nile red fluorescence staining. The plots beside the images show the quantitation analysis of neutral lipids fluorescence intensity. Data shown in each plot are the mean ± SD of three independent experiments.

To elucidate the molecular mechanism by which SIP might have alleviated UVB-induced skin dryness, HaCaT cells were treated with SIP in the presence of a specific PPAR-α inhibitor (GW6471) and then exposed to UVB irradiation (200 mJ/cm^2^)*.* After blocking PPAR-α, the protein levels of ABCA1 or the mRNA levels of ELOVL1, ELOVL4, and ELOVL6 in the SIP-treated HaCaT cells did not increase, but the levels of filaggrin and involucrin still increased (Figures 7A, B). Furthermore, blocking PPAR-α also resulted in the loss of the ability of SIP to prevent the UVB-induced decrease in neutral lipid (Figure 7C). Thus, it appeared that the protective effect of SIP against UVB-induced inhibition of keratinocyte differentiation and lipid synthesis might be entirely or partially dependent on the activation of PPAR-α.

### 3.8 PPAR-α is involved in the protective effects of SIP

**FIGURE 8:**
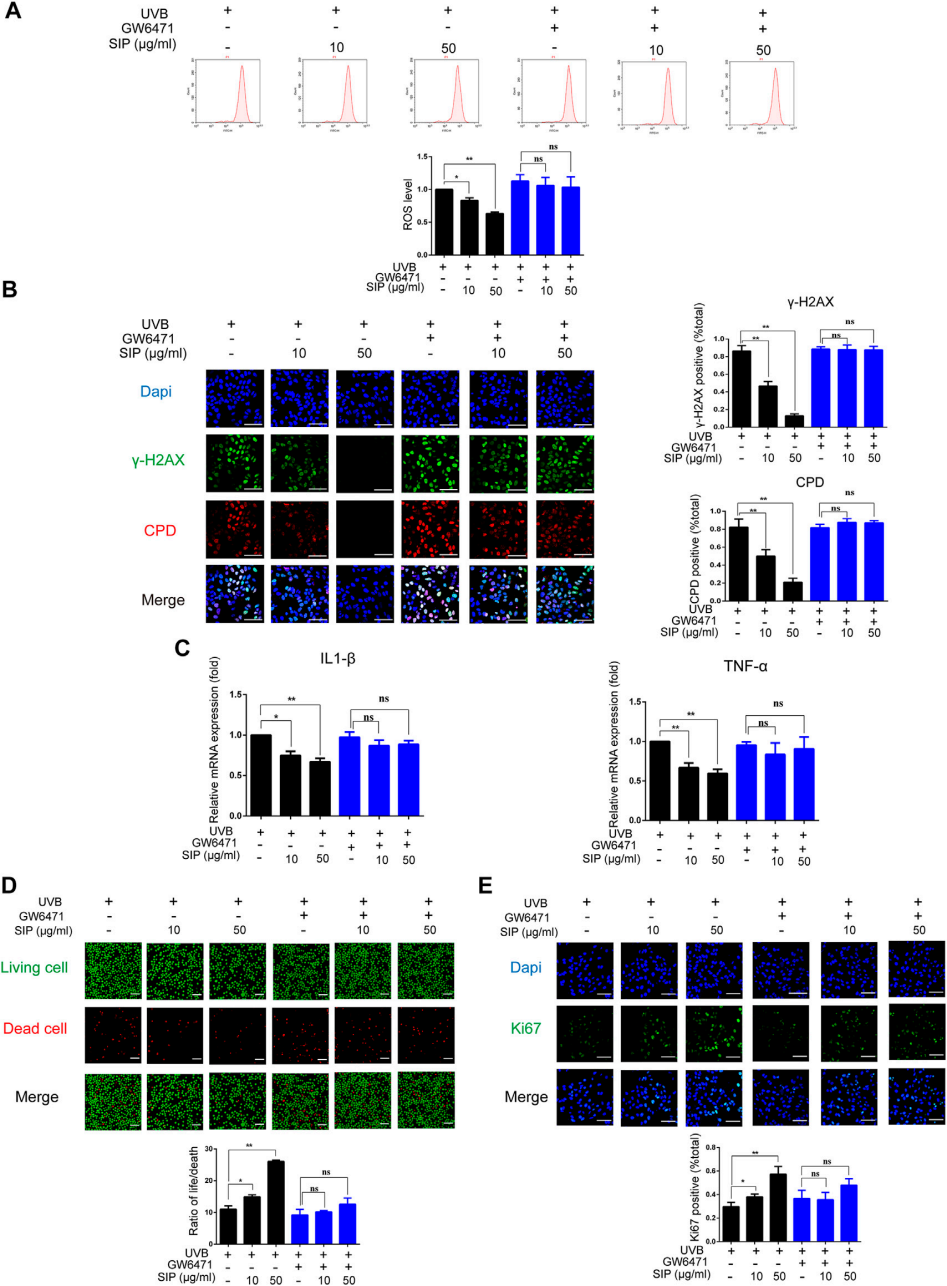
PPAR-α is involved in the protection of SIP against oxidative-stress damage induced by UVB in HaCaT cells. **(A)** Detection of ROS levels in HaCaT cells by flow cytometry. The plots under the images show the quantitation analysis of ROS levels. **(B)** Changes in *γ*-H2AX and CPD levels in HaCaT cells as examined by immunofluorescence staining. The plots beside the images show the quantitation analysis of *γ*-H2AX and CPD positive cells. **(C)** Changes in the expression levels of pro-inflammatory factors (IL-1β and TNF-α) in HaCaT cells as measured by qPCR. **(D)** Changes in HaCaT cell death as examined by Calcein-AM/PI fluorescence double staining. The plots below the images show the ratio of life and dead cells. **(E)** Changes in the proliferation marker Ki67 in HaCaT cells as examined by immunofluorescence staining. The plots below the images show the quantitation analysis of Ki67 positive cells. Data shown in each plot are the mean ± SD of three independent experiments. “*” and “**” indicate significantly different from the UVB only at the *p* < .05 and *p* < .01 levels respectively, in no GW6471 treatment group.

Through the action of PPAR-α, SIP could regulate the differentiation of keratinocytes and the disorder of epidermal intercellular lipid synthesis in UVB-irradiated HaCaT cells. To further investigate the role of PPAR-α in the protective effect of SIP against UVB-induced skin dryness, HaCaT cells were treated with SIP in the presence of a specific PPAR-α inhibitor (GW6471) and then exposed to UVB irradiation (200 mJ/cm^2^). These cells displayed no increase in ROS production or *γ*-H2AX and CPD contents as shown by flow cytometry analysis and immunofluorescence analysis, respectively (Figures 8A, B). Besides, in the presence of GW6471, SIP treatment did not reduce the mRNA levels of IL-1β and TNF-α in UVB-irradiated HaCaT cells (Figure 8C). SIP also did not reduce the number of dead cells increased by UVB irradiation or increase the number of Ki67-labeled cells decreased by UVB irradiation in the presence of GW6471 as confirmed by calcein-AM/PI fluorescence and Ki67 fluorescence staining (Figures 8D, E). The findings suggested that the involvement of PPAR-α in the anti-oxidative and anti-inflammatory activities of SIP might form an important mechanism by which SIP could reduce the extent of UVB-induced cell death.

## 4 Discussion

Dryness is a symptom of epidermal barrier dysfunction (Fischer et al., 2001). Our data confirmed the alleviation of oxidative stress and DNA damage mediated by SIP in UVB-irradiated HaCaT cells and suggested that SIP could attenuate the secondary inflammatory response by effectively inhibiting the expression of the pro-inflammatory factors IL-1β and TNF-α in UVB-induced HaCaT cells. This would indicate that SIP might prevent further damage to the epidermal barrier following UVB exposure by inhibiting the onset of UVB-induced oxidative stress*.* Furthermore, SIP could prevent the excessive proliferation of epidermal cells, SIP would reduce UVB-induced epidermal hyperplasia and restore cellular homeostasis. Neutral lipids constitute the majority of intercellular lipids, and their metabolism is required for the epidermal permeability barrier to function properly. Since SIP could reverse UVB-induced inhibition of neutral lipid synthesis in keratinocytes, it might imply that the protective effect of SIP against UVB-induced skin dryness was also dependent on neutral lipids in the stratum corneum. However, how SIP could enforce this role remains to be explored. Notably, SIP reversed the reduction of lipid transporter ABCA1 and increased the expression of the lipid chain elongation enzymes ELOVL1, ELOVL4, and ELOVL6 in UVB-irradiated HaCaT cells (Figure 6). Shortening the chain length of the lipid molecules can lead to abnormal alterations in the intercellular lipid structure of the stratum corneum, resulting in the loss of epidermal barrier function. Ceramide is one of the most important lipid components of the skin permeability barrier, and the chain length of fatty acid groups strongly influences the function of ceramides, with the C30-C36-hydroxy fatty acid type known as acyl ceramide required for skin barrier constitution (Hirabayashi et al., 2019). ELOVLs are key enzymes in the synthesis of long-chain fatty acids, with ELOVL1, ELOVL4, and ELOVL6 being critical for ensuring normal epidermal barrier function (Mueller et al., 2019; Zhu et al., 2019). ELOVL1 and ELOVL4 are sequentially involved in the lengthening of the fatty acid fraction: ELOVL1 lengthens long-chain acyl coenzyme A to C26, and then ELOVL4 lengthens it to C30-C36. Short-chain (C16) fatty acids synthesized in the cytoplasm must be converted to long-chain (C18) fatty acids by ELOVL6 before ceramide synthase can catalyze their formation into long-chain ceramides (Shimamura et al., 2009). Keratinocytes require abundant cholesterol for cutaneous permeability barrier function and in keratinocytes cholesterol exists in the form of lamellar bodies. ABCA1 is a cell membrane transporter protein that is widely expressed in many tissues and is involved in cholesterol homeostasis by regulating intracellular cholesterol efflux (Jacobo-Albavera et al., 2021). As a result, ABCA1 plays an important role in the formation of lamellar bodies. PPAR-α activation promotes lipid synthesis and increases ABCA1 expression, which enhances epidermal permeability barrier function (Jiang et al., 2006). UVB radiation inhibits the fatty acid elongation enzymes (ELOVL1, ELOVL4, and ELOVL6) and lipid transporter protein (ABCA1), resulting in shorter chain-length fatty acids and ceramides in the epidermis. Therefore, we hypothesize that SIP may prevent UVB-induced epidermal lipid synthesis disorder by protecting the synthesis of long-chain fatty acids and ceramides to promote the synthesis of neutral lipids. The association between PPAR-α activation and the protective effect of SIP against UVB-induced damage constituted another interesting finding of our study. PPARs are members of the nuclear hormone receptor (NHR) family that when activated by the binding of ligand and regulating the transcription of target genes and exert their biological effects. Studies conducted by Schmuth and Man indicate that all PPAR isoforms (PPAR-α, PPAR-β/δ, PPAR-γ) can promote epidermal cell differentiation, inhibit epidermal cell proliferation, and enhance skin barrier function (Man et al., 2006; Schmuth et al., 2014). Our data also revealed the regulation of PPAR-α protein expression by SIP but no effect of SIP on PPAR-γ expression was observed. LXRs are another class of ligand-activated nuclear receptors that have been shown to increase the expression of keratinocyte differentiation-related genes, SIP treatment had no significant effect on LXR expression (Figure 4), suggesting that the protective effect of SIP against UVB may be mediated by PPAR-α. PPARs can also suppress the inflammatory response by promoting NF-κB inactivation and reducing the amount of ROS (Huang et al., 2016; Korbecki et al., 2019), the anti-inflammatory effect of SIP may be attributed to PPAR-α-mediated inactivation of NF-κB. Transcriptome analysis revealed that PPAR is required for partial regulation of the ELOVL1 gene and that regulated genes contain PPAR-α and PPAR-γ binding motifs in their 5′-regions (Mueller et al., 2019). PPAR-α also positively regulates the transcription of ELOVL4 and ELOVL6 (Rogue et al., 2011; Zhu et al., 2019). UVB radiation inhibited PPAR-α and PPAR-α target gene expression. UVB can reduce lipid content in the epidermis by reducing lipid synthesis-related enzymes such as acetyl-CoA carboxylase (ACC), fatty acid synthase (FAS), stearoyl-CoA desaturase (SCD), and sterol regulatory element binding proteins (SREBPs) (Kim et al., 2010). Because UVB radiation reduces neutral lipids, which include free fatty acids in the epidermis, the level of free fatty acid expression in the epidermis is altered, which regulates PPAR-α transcription. SIP restored the expression levels of PPAR-α and PPAR-α target genes (Figure 7). Further studies indicated that inhibiting PPAR-α completely abolished the protective effect of SIP against UVB-induced epidermal cell damage (Figure 8). This partially validated our previous hypothesis that the protective effect of SIP against UVB-induced skin dryness is activated by PPAR-α. Notably, PPAR-α could be partially involved in SIP-mediated regulation of HaCaT cell differentiation. SIP might promote keratinocyte differentiation through other elements such as calcium-sensitive receptors (CaSR), Wnt/β-catenin, Notch, and MAPK signaling pathways (Popp et al., 2014). Further research is required to determine whether SIP can regulate the proliferation and differentiation of keratinocytes *via* these ways, and whether SIP would provide this protection to the epidermis by alleviating the extent of UVB-induced epidermal damage or by promoting the repair of UVB-induced epidermal damage.

## 5 Conclusion and prospects

**FIGURE 9:**
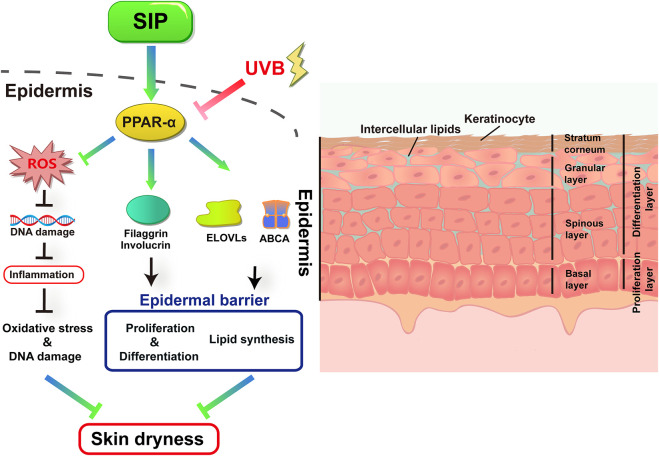
Proposed protective mechanism of SIP in its protection against UVB-induced skin dryness. UVB irradiation inhibits the transduction of the PPAR-α pathway in the epidermis. UVB-induced excessive ROS damages cellular DNA and causes secondary inflammatory responses. UVB further disrupts epidermal barrier function by inhibiting the expression of keratinocyte differentiation factors filaggrin and involucrin, as well as lipid synthesis and transport-related factors ELOVLs and ABCA1, resulting in skin dryness. SIP can protect skin from UVB by activating the PPAR-α pathway.

We have presented here for the first time that SIP could alleviate UVB-induced oxidative stress in keratinocytes and inhibit the inflammatory response, and activate the PPAR-α pathway, which would directly impact the proliferation and differentiation of keratinocytes and mitigate lipid synthesis disorder. The result was an enhancement in the epidermal barrier function for maintaining skin hydration and preventing UVB-induced skin dryness (Figure 9). These findings could provide a theoretical and experimental basis for the development and application of SIP in protecting against UVB-induced skin dryness.

## Data Availability

The original contributions presented in the study are included in the article/supplementary materials, further inquiries can be directed to the corresponding authors.
